# Editorial: Targeting nucleotide metabolism for enhancing antitumor immunity

**DOI:** 10.3389/fimmu.2024.1412057

**Published:** 2024-04-23

**Authors:** Jun Wu, Yu Rong, Tian Li, Cornelia M. Wilson, Yazhou He, Danqian Chen, Jin Han, Xingmei Zhang

**Affiliations:** ^1^Division for Research, Shandong College of Traditional Chinese Medicine, Yantai, China; ^2^Department of Thoracic Surgery, The First Affiliated Hospital of Hebei North University, Zhangjiakou, China; ^3^School of Basic Medicine, Fourth Military Medical University, Xi'an, China; ^4^School of Psychology and Life Sciences, Life Sciences Industry Liaison Lab, Canterbury Christ Church University, Sandwich, United Kingdom; ^5^West China School of Public Health and West China Fourth Hospital, Sichuan University, Chengdu, China; ^6^School of Life Sciences, Northwest University, Xi’an, China; ^7^Department of Nephrology, Chang’an District Hospital, Xi’an, China; ^8^College of Medical Technology, Chengdu University of Traditional Chinese Medicine, Chengdu, China

**Keywords:** nucleotide metabolism, nucleotide, oncoimmunity, cancer, neoplasms

Neoplasm remain the main killer worldwide (1–5).Tumor microenvironment (TME) is a special environment for tumor survival, which has the characteristics of acidity, lack of nutrition and immunosuppression (6). Among them, the competition and cooperation of tumor, fibroblast, endothelial cell, immune cell and other clonal populations play a key role in cell proliferation, tumor metastasis and the development of therapeutic resistance (7). Nucleotides are major components of genetic material and are essential for DNA and RNA biosynthesis, cell signaling, enzyme regulation, and metabolism (8). Malignancies exhibit rapid growth and rely more on *de novo* synthesis than external sources (9), with very high nucleotide requirements. Immune cells in the TME, especially effector T cells, require rapid expansion when the immune response is activated, as well as lots of nucleotides in order to replicate (10). The regulation of TME and immune cell function is strongly dependent on nucleotide metabolism, according to a growing body of research (6, 11).

LncRNAs (long non-coding RNAs) are a class of RNAs longer than 200 nucleotides that play a variety of roles in cell differentiation, cell cycle regulation, and epigenetic regulation. Ye et al. explored the nucleotide molecular mechanism of compound Fushen injection in the treatment of lung adenocarcinoma and found that mir-15b could enhance the proliferation and migration of lung adenocarcinoma by targeting BCL2, and increase the expression of BIRC5 could improve the radiotherapy efficacy of LUAD cells. Copper plays an important role in various processes related to nucleotide metabolism, especially ribonucleotide reductase is a key enzyme affected. It is an important electron carrier for ribonucleotide reductase and is essential for maintaining appropriate nucleotide levels and ensuring accurate DNA replication and repair. Luo et al. found that IGFBP2, a biomarker associated with copper metabolism, is not only a glioma tumor promoter that promotes disease progression, but also affects immunotherapy response. Zhao et al. used TCGA and XENA-TCGA databases to find that lncRNA SLC25A25-AS1 is highly expressed in many cancers. Further analysis of clinical samples by prostate cancer (PC) showed that high expression of SLC25A25-AS1 was associated with T stage, clinical stage, Gleason score (GS) and poor prognosis. SLC25A25-AS1 is associated with an infiltration of CD8 T cells, IDCs (interstitial dendritic cells), macrophages, and other immune cells in the PC immune microenvironment. Targeting SLC25A25-AS1 in conjunction with PC immune microenvironment provides a potential therapeutic target for the treatment of PC.

Zhang et al. found that lung adenocarcinomatosis with lower types of immune cell infiltration were more responsive to immune checkpoint (LAG3, PD-L1, IDO1) inhibition and targeted drugs (JNK inhibitor VIII, BI-D1870, RO-3306, etc.). Exonuclease 1 (EXO1) genes encodes a protein with 5′ to 3′ exonuclease activity as well as RNase H activity. It has both an endonuclease domain and an exonuclease domain. Capable of acting on nucleotide excision repair (NER) and mismatch repair (MMR) pathways (12). In addition, it was also found that EXO1, one of the key module genes, is related to a variety of immune cells in the immune environment and significantly affects anti-tumor immunity (Zhang et al.). Autophagy is an intracellular lysosomal degradation pathway that reconnects cellular metabolism. Metabolites produced by this process can be reused for core biosynthetic processes or energy production, including nucleotides (13). Another study used bioinformatics methods to identify immune heterogeneity in hepatocellular carcinoma (HCC) from public databases and analyze genes associated with autophagy. In malignant tumors, autophagy-related pathways were more prevalent, and group C1 was associated with a better survival rate, enhanced immune infiltration, and a greater response to immunotherapy (Liu et al.).

Zhang et al. also found that immune cells in the tumor microenvironment were closely related to the effect of tumor immunotherapy and chemotherapy. Patients with a high degree of infiltration of antigen presenting B cells, tumor killer natural killer cells, CD8+T cells and other cells in the immune microenvironment have enhanced immune status, which is more suitable for immunotherapy and chemotherapy. Copper plays an important role in various processes related to nucleotide metabolism, especially ribonucleotide reductase is a key enzyme affected. It is an important electron carrier for ribonucleotide reductase and is essential for maintaining appropriate nucleotide levels and ensuring accurate DNA replication and repair. Luo et al. found that IGFBP2, a biomarker associated with copper metabolism, is not only a glioma tumor promoter that promotes disease progression, but also affects immunotherapy response.

Studies have shown that aberrant nucleotide metabolism not only accelerates tumor development, but also suppresses normal immune responses (14–17). Studies have demonstrated that targeting nucleotide metabolism can increase anti-tumor immune responses through multiple pathways (8, 9). Such as activating the host immune system by maintaining the concentration of several important metabolites, such as adenosine and ATP; The imbalance of purine/pyrimidine ratio leads to an increase in tumor mutation burden (TMB), which leads to an increase in surface neoantigen expression, thus enhancing immunotherapy. According to Keshet and colleagues, inhibiting purine synthesis increases the ratio of pyrimidines to purines, improves immunoproteasome expression, and improves anti-PD-1 response in autologous primary T cells (18). Thus, targeting nucleotide metabolism in the TME holds great promise for preventing and treating cancer.

Methotrexate was the earliest antimetabolite used to treat cancer. In cancer chemotherapy, more than 20 approved nucleotides and nucleotide analogues are used. These drugs account for nearly 20% of all cancer therapies (8). It is not well understood how other cell types influence metabolic cancer therapy in the context of the tumor microenvironment. Hyrossova et al. and Youssef et al. in the manuscript described the effects of interventions including targeting nucleotide pathways on the cellular components of the tumor microenvironment (9, 10).

Drugs that target the adenosine pathway are shown to transform an immunosuppressive microenvironment into a more immunopermissive one and reduce metastasis and resistance to treatment (14). The review by Sek (19), Xia (20) et al. provides a detailed description of the role of adenosine and its receptor signaling in tumors and immune cells. CD73, CD39 and CD38 expressed on tumor cells and immune cells catabolize extracellular ATP into adenosine (ADO) and accumulate in TME, thus mediating the suppression of anti-tumor immunity through the activation of ADO receptors(A1R、A2AR、A2BR、A3R). The α subunits of G proteins are classified into 4 major families (Gαs, Gαi/o, Gq/11, Gα12/13), which are the main signaling pathways of the A2AR, A2BR and A1R, A3R receptors, and bind to the Gαs and Gαi/o family of Gα proteins, respectively. A2AR and A2BR conjugated to Gαs can promote cyclic adenosine monophosphase (cAMP) accumulation and play a dominant role in suppressing anti-tumor immune cell responses. In contrast, A1R and A3R conjugated to Gαi/o subunits inhibited the production of adenylyl cyclase and cAMP (19). Targeting the CD39/CD73/A2AR signaling pathway shows powerful anti-tumor efficacy, which predicts its wide application in the clinical field (21). In addition, Franco et al. believe that A2AR is not only the main target of immunotherapy methods, A2BR seems to be more promising as a target for chemotherapy intervention (or even radiotherapy) (22). It is well known that many malignant tumors overexpress purinergic receptors, which is associated with tumor cell proliferation, metastasis, therapeutic resistance and poor prognoses. Jia et al. and Aria et al. described in the review that P2X7R inhibition has a powerful effect in reversing therapeutic resistance in various types of cancer therapy (23, 24).

A tumor microenvironment, including immune cells, fibroblasts, endothelial cells, and fibroblasts, is involved in the development of radioresistance, among other factors. A single metabolic target is not enough to overcome tumor cell radioresistance due to metabolic heterogeneity. According to Yu et al., related nucleotide synthesis enzymes (such as TS/PPAT/IMPDH) have been shown to be radiation sensitizing (25).

A salvage pathway has nevertheless been demonstrated to be associated with cancer development, even though immune cells and cancer cells prefer *de novo* nucleotide synthesis (26). Walter et al. proposed targeting the pyrimidine rescue pathway in DNA replication alone or in combination with inhibitors of *de novo* pyrimidine synthesis to overcome the limitations of commonly used antimetabolites in various preclinical cancer models and clinical trials (20). For the purpose of overcoming the limitations of commonly used antimetabolites in various preclinical cancer models clinical trials. Walter et al. proposed targeting the pyrimidine rescue pathway in DNA replication alone or in combination with inhibitors of *de novo* pyrimidine synthesis.

Overall, given the particularly high proliferation rate and active nucleotide synthesis of cancer cells, interfering with nucleotide metabolism has been proposed as a promising strategy for antitumor therapy. The complexity and diversity of interactions between tumor cells and immune cells in relation to their intracellular and extracellular nucleotide metabolisms necessitates more personalized approaches.

## Author contributions

JW: Writing – original draft. YR: Writing – original draft. TL: Writing – original draft. CW: Writing – original draft. YH: Writing – review & editing. DC: Writing – review & editing. JH: Writing – review & editing. XZ: Writing – review & editing.
